# Gut Commensal-Induced IκBζ Expression in Dendritic Cells Influences the Th17 Response

**DOI:** 10.3389/fimmu.2020.612336

**Published:** 2021-01-19

**Authors:** Lena Michaelis, Marcel Treß, Hanna-Christine Löw, Johanna Klees, Christian Klameth, Anna Lange, Anne Grießhammer, Andrea Schäfer, Sarah Menz, Alex Steimle, Klaus Schulze-Osthoff, Julia-Stefanie Frick

**Affiliations:** ^1^ Department for Medical Microbiology and Hygiene, Interfaculty Institute for Microbiology and Infection Medicine, University of Tübingen, Tübingen, Germany; ^2^ Department of Infection and Immunity, Luxembourg Institute of Health, Esch-sur-Alzette, Luxembourg; ^3^ Interfaculty Institute for Biochemistry, University of Tübingen, Tübingen, Germany

**Keywords:** dendritic cells, Th17, intestinal commensals, inflammatory bowel disease, immunogenicity, *Escherichia coli*, *Bacteroides vulgatus*, IκBζ

## Abstract

Intestinal commensal bacteria can have a large impact on the state of health and disease of the host. Regulation of Th17 cell development by gut commensals is known to contribute to their dichotomous role in promoting gut homeostasis and host defense, or development of autoimmune diseases. Yet, the underlying mechanisms remain to be fully elucidated. One candidate factor contributing to Th17 differentiation, and the expression of which could be influenced by commensals is the atypical nuclear IκB protein IκBζ. IκBζ acts as a transcriptional regulator of the expression of Th17-related secondary response genes in many cell types including dendritic cells (DCs). Insights into the regulation of IκBζ in DCs could shed light on how these immune sentinel cells at the interface between commensals, innate and adaptive immune system drive an immune-tolerogenic or inflammatory Th17 cell response. In this study, the influence of two gut commensals of low (*Bacteroides vulgatus*) or high (*Escherichia coli*) immunogenicity on IκBζ expression in DCs and its downstream effects was analyzed. We observed that the amount of IκBζ expression and secretion of Th17-inducing cytokines correlated with the immunogenicity of these commensals. However, under immune-balanced conditions, *E. coli* also strongly induced an IκBζ-dependent secretion of anti-inflammatory IL-10, facilitating a counter-regulative Treg response as assessed in *in vitro* CD4^+^ T cell polarization assays. Yet, in an *in vivo* mouse model of T cell-induced colitis, prone to inflammatory and autoimmune conditions, administration of *E. coli* promoted an expansion of rather pro-inflammatory T helper cell subsets whereas administration of *B. vulgatus* resulted in the induction of protective T helper cell subsets. These findings might contribute to the development of new therapeutic strategies for the treatment of autoimmune diseases using commensals or commensal-derived components.

## Introduction

With an area around 200 times larger than the skin, the gastrointestinal mucosa is the largest immunological organ in the body (1). It faces a challenging environment and needs to maintain a careful balance between fighting intestinal intruders and tolerating commensal and nutrition-derived antigens (2). Failure of maintaining gut homeostasis promotes a shift in the microbiota composition, known as dysbiosis and characterized by a loss of bacterial diversity and/or commensals, as well as a bloom of pathobionts (3). A dysbiotic microbiota has been associated with many multifactorial autoimmune diseases such as multiple sclerosis, type 1 diabetes mellitus and inflammatory bowel diseases (IBD) (2, 4).

Dendritic cells (DCs) play a major role in the regulation of gastrointestinal mucosal immunity since they are among the first-line antigen-presenting cells at mucosal surfaces and link the innate and the adaptive immune system (5). DCs encounter a diversity of gut microbes and respond by inducing either immune tolerance to harmless commensal-derived antigens or an inflammatory response to potential pathogens. DCs recognize various surface structures on bacteria, so-called microbe-associated molecular patterns (MAMPs), *via* their patter recognition receptors (PRRs), such as Toll-like receptors (TLRs) (6, 7). Upon sampling of these antigens, DCs undergo a differentiation process resulting in e.g., semi-mature (smDCs) or mature DCs (mDCs), characterized by low or high expression of activation and maturation markers, respectively (8, 9). Under homeostatic conditions, DCs orchestrate the differentiation of naïve CD4^+^ T cells into functionally distinct T helper cell subsets by creating an environmental cytokine milieu required for the balanced co-existence of regulatory and inflammatory CD4^+^ T cells (10). In this role, smDCs are known to induce T cell anergy and regulatory T cells (Tregs) whereas mDCs are potent antigen presenting cells promoting CD4^+^ and CD8^+^ T cell responses (9).

A subset of IL-17-secreting CD4^+^ T cells (Th17 cells) plays a dichotomous role in gut homeostasis by promoting protection against fungal and bacterial pathogens on one side, and driving inflammatory pathology and development of autoimmune diseases on the other side (11, 12). The orphan nuclear receptor RORγt is the lineage-determining “master” transcription factor directing the production of the hallmark cytokines IL-17A, IL-17F as well as IL-21 and IL-22 (12, 13). Among these, especially IL-17A plays a dominant role in driving autoimmunity (13). Due to intrinsic instability and plasticity, Th17 cells are able to transdifferentiate to more inflammatory or regulatory phenotypes in response to fluctuating physiological environments (10, 12). Differentiation of Th17 cells is dependent on interleukin 6 (IL-6) and transforming growth factor β (TGFβ), whereas their full maturation depends on IL-1β and IL-23, possibly favoring their pathological activity in the induction of autoimmunity (14, 15). Beyond their demonstrated ability to secrete all these cytokines, how DCs influence plasticity and poise protective and inflammatory responses is not fully known (14).

Besides RORγt, another transcription factor required for Th17 development is the atypical inhibitor of the nuclear factor κB (IκB) protein IκBζ which harbors six ankyrin repeats at its carboxyl terminus, and is encoded by the *Nfkbiz* gene (16, 17). Also known as MAIL or INAP, IκBζ is expressed in a variety of cell types and is essential for the induction of a subset of secondary response genes, e.g., *Il-6*, *Il-12*, *Il-17*, and *Ccl2* (16, 18–20). Transcription of the *Nfkbiz* gene is rapidly induced as primary NFκB response gene upon TLR- and cytokine-receptor signaling (18, 19, 21). The necessity of IκBζ in Th17 development was shown in *Nfkbiz* ^-/-^ mice which were resistant to experimental autoimmune encephalomyelitis (EAE), a model of Th17-mediated autoimmune disease with a multiple sclerosis-like phenotype (16, 22).

IκBζ expression in DCs is of particular importance for regulating Th17 development due to the steering role of DCs in states of homeostasis and inflammation. Yet, the impact of a shifting microbiota, as it is observed in states of inflammatory and autoimmune diseases, on IκBζ-dependent, DC-mediated T cell differentiation has not been characterized. In this study, we elucidate the impact of two model gut commensals on the induction of Th17 responses mediated by DCs. We could show that the induction of IκBζ-expression by commensals with low (*Bacteroides vulgatus*) and high (*Escherichia coli*) immunogenicity positively correlates with their immunogenicity in DCs. Furthermore, in an *in vivo* mouse model of IBD, enhancing abundance of these commensals influenced the differentiation of intestinal T helper cells towards rather protective and regulatory phenotypes (*B. vulgatus*) or pro-inflammatory phenotypes (*E. coli*). This effect could experimentally be traced back to the differential expression of IκBζ in DCs.

## Materials and Methods

### Bacteria

*Escherichia coli* mpk (23) was grown overnight in Luria-Bertani (LB) medium under aerobic conditions at 37°C and subcultivated in the same medium for 2.5 h the next day prior quantification to ensure logarithmic growth phase. *Bacteroides vulgatus* mpk (23) was cultivated in liver broth for 3 days and, prior to quantification, subcultivated in Brain-Heart-Infusion (BHI) medium for 2 days and anaerobic conditions at 37°C to ensure logarithmic growth phase.

### Mice

Female C57BL/6NCrl (WT) mice were purchased from Charles River Laboratories. C57BL/6J-Rag1^tm1Mom^ (*Rag1* ^-/-^), *TLR2^-/-^*, *TLR4* ^-/-^ and *TLR2* ^-/-^x*TLR4* ^-/-^ mice were obtained from Jackson Laboratories. *Nfkbiz* ^-/-^ mice were kindly provided by Dr. M Morimatsu (24) and bred from *Nfkbiz* ^+/-^ breeding pairs. For isolation of bone marrow and T cells, 6–12 week old mice were used. All mice were kept and bred under specific pathogen-free (SPF) conditions.

### Cultivation and Stimulation of Bone Marrow-Derived Dendritic Cells

Bone marrow cells were isolated and cultivated as described previously (25). At day 7 after isolation, CD11c^+^ bone marrow derived-dendritic cells (BMDCs) were used for *in vitro* experiments. 1 × 10^6^ BMDCs/ml were stimulated with PBS (mock, Thermo Fisher Scientific), *B. vulgatus* or *E. coli* at a multiplicity of infection (MOI) of 1 at 37°C. 100 ng/ml isolated LPS of *B. vulgatus* [LPS_BV_, isolated as described in (26, 27) and (28)] or *E. coli* [LPS_EC_, isolated as described in (26, 27) and (28)] were used for stimulation. For stimulation with a complex microbiota, fecal samples were collected from SPF *Rag1^-/-^* mice prior to administration of bacteria and induction of colitis, as well as at the end of the experiment (see T cell transfer in *Rag1^-/-^* mice). Samples were weighed, dissolved in sterile PBS to a stock concentration of 50 mg/ml, heat-inactivated for 15 min at 80°C and filtered through a 100 µm cell sieve. Fecal samples were then further diluted in sterile PBS and BMDCs were stimulated with prepared fecal samples in a concentration of 100 µg/ml. Gentamicin (1 µg/ml) was added to all samples in order to prevent bacterial overgrowth and to create equal treatment conditions. Cells were harvested after the indicated stimulation periods and processed for subsequent analyses.

### Cultivation and Stimulation of mICcl2 Cells

Trans-immortalized mouse intestinal epithelial cells derived from the small intestine of a transgenic mouse were cultured as described elsewhere (29). One day prior stimulation, mICcl2 cells were seeded at a concentration of 5 × 10^5^ cells/ml. Cells were stimulated with PBS (mock), *B. vulgatus* or *E. coli* at a MOI of 10 for 2 h and gentamicin (1 µg/ml) was added to all samples in order to prevent bacterial overgrowth and to ensure equal treatment conditions. Cells were gently detached with 0.05% trypsin-EDTA (Gibco) from culture vessels and processed for further analysis.

### Isolation of Naïve T Cells

For adoptive T cell transfer and *in vitro* T cell polarization assay, splenic naïve CD3^+^CD4^+^CD25^-^CD45RB^+^ T cells from female WT mice were purified using a MACS-based negative selection kit (Miltenyi) according to the manufacturer’s instructions.

### T Cell Polarization Assay

Antigen-independent activation of naïve CD4^+^ T cells occurred by overnight incubation with plate-bound anti-CD3 (145-2C11) antibodies (BioLegend, coated with 10 µg/ml in PBS) and 2 µg/ml anti-CD28 (37.51) (BioLegend). As polarizing factor, sterile-filtrated cell culture supernatants of 16 h-stimulated BMDCs containing stimulus-dependent cytokine concentrations were used, diluted 1:2 in T cell medium (RPMI 1640 supplemented with 10% FCS, 50 μM 2-mercaptoethanol, 25 mM HEPES, 1% non-essential amino-acids, 1% sodium pyruvate and 1% penicillin/streptomycin). In order to mimic an imbalanced cytokine milieu, neutralizing anti-IL-10 (JES5-2A5) antibodies (BioLegend, 10 µg/ml) were added to naïve T cells simultaneously with the BMDC supernatant. Cells were incubated for 4 h with GolgiStop (BD) prior to end of polarization time and processed for flow cytometry analysis.

### Dextran Sodium Sulfate (DSS)-Induced Colitis in WT and *Nfkbiz* ^-/-^ Mice

Acute DSS colitis was induced in SPF WT and *Nfkbiz* ^-/-^ mice by administration of 2.5% (w/v) DSS (molecular weight 36–50 kDa, MP Biomedicals) dissolved in drinking water for 7 days. Onset of inflammation was assessed on day 0 and on days 3–7 by monitoring body weight and disease activity index (DAI) with parameters ranging from 0–3 regarding blood in stool and on anus, stool consistency, relieving posture and appearance of fur. Colon tissue was used for histopathological analysis by fixing it in 4% formalin and sections stained with Hematoxylin/Eosin (H&E).

### T Cell Transfer Colitis in Rag1-/- Mice

Administration of *B. vulgatus* or *E. coli* to 10 week-old *Rag1* ^-/-^ mice *via* drinking water in a concentration of 2 × 10^8^ bacteria/ml started one week prior to intraperitoneal injection of 5x10^5^ naïve T cells. Replacing drinking water with bacteria and weighing of mice occurred twice a week. Mice were kept in IVCs in order to maintain stability of the newly developed microbiota composition. Fecal samples were collected prior to administration of bacteria and at the end of the experiment. Mice were sacrificed 5 weeks after T cell transplantation for analysis. Degree of colonic inflammation was determined using colonic histological sections, stained by H&E and scored as described elsewhere (30).

### Isolation of Dendritic Cells and T Cells From Colonic Lamina Propria and Mesenteric Lymph Nodes

For isolation of colonic lamina propria (cLP) cells, caecum and colon were thoroughly washed with PBS and cut into 1.5 cm pieces, followed by two incubation periods in HBSS/5% FCS/2 mM EDTA/1 mM DTT, washing in HBSS/5% FCS/1 mM HEPES for 10 min and digestion of minced pieces in RPMI/40 U/ml DNase I/0.12 mg/ml collagenase for 15 min. All steps were performed at 37°C and gentle stirring, with vortexing and filtering through a 100 µM cell strainer in between single steps. Final cell suspension was washed twice with ice-cold HBSS/5% FCS. Immune cells from mesenteric lymph nodes (mLN) were isolated by gentle disruption and passing through a nylon cell strainer (40 µm mesh) with PBS/1% FCS and a washing step with PBS/1% FCS. T cells from cLP and mLN were activated with leukocyte activation cocktail (BD Biosciences) for 4 h with subsequent processing for flow cytometry analysis.

### RNA Isolation and RT-PCR

Isolation of RNA from mICcl2 cells, BMDCs and colonic tissue lysates was performed using Qiagen’s RNeasy Mini Kit according to manufacturer’s instructions. Additional DNA digestion was conducted by using the DNA-free DNA Removal Kit (Thermo Fisher Scientific). SybrGreen based quantitative RT-PCR was performed on a Roche LightCycler480 using Qiagen SybrGreen RT-PCR Kit. Primer annealing occurred at 60°C. 10–100 ng DNase-digested RNA was used for qRT-PCR. Relative mRNA expression in cells stimulated with bacteria to unstimulated cells was determined by using β-actin as housekeeping gene according to the ΔΔCp-method, which takes into account the specific amplification efficiency of every primer pair and each PCR run. Primer sequences: *Nfkbiz* (NCBI Gene ID: 80859) forward: GTGGGAGAACAGATCCGACG, reverse: AGTGAGTGTCGCTGAACCAG; β-actin (NCBI Gene ID: 11461) forward: CCCTGTGCTGCTCACCGA, reverse: ACAGTGTGGGTGACCCCGTC.

### Quantification of Bacteria in Fecal Samples

Plasmid standards were generated by blunt-end cloning using pJET (Thermo Fisher Scientific) and the respective specific 16s PCR fragments of *E. coli* (Primer forward: GTTAATACCTTTGCTCATTGA, reverse: ACCAGGGTATCTAATCCTGTT (31) or *B. vulgatus* (Primer forward: AACCTGCCGTCTACTCTT, reverse: CAACTGACTTAAACATCCAT (32). The concentration of the isolated plasmids was determined and the standard concentrations were prepared in 10-fold serial dilutions in a range of 100,000–10 copies. Bacterial DNA was isolated using the QIAamp DNA Stool Mini Kit (Qiagen) according to manufacturer’s instructions. DNA concentration was measured using Qubit dsDNA High Sensitivity Assay (Thermo Fisher Scientific). For the qPCR measurement, DNA concentrations were adjusted to 5 ng per reaction, and PCR was performed using QuantiFast SYBR Green PCR Kit (Qiagen). Bacterial copy numbers were determined by a standard curve. For this purpose, log10 of standard copy numbers were plotted against ct-values.

### Cytokine Analysis

For determination of IL-6, IL-10, IL-23, IL-1β concentrations in cell culture supernatants, ELISA kits purchased from BD Biosciences or eBiosciences were used according to manufacturers’ instructions. For detection of mouse serum cytokines, the Bio-Plex Pro assays Mouse Cytokine 23-Plex and sets for Mouse IL-17F, Mouse IL-21, Mouse IL-22, Mouse IL-23 and TGFβ1 (Bio-Rad) were performed according to manufacturer’s instruction and analyzed on a Bio-Plex 200.

### Flow Cytometry Analysis

After harvesting or isolation, mICcl2cells, BMDCs, cLP cells and mLN cells were washed and Fc-receptors were blocked for 15 min at 4°C. Staining with fixable viability dyes (Thermo Fisher Scientific) for 15 min at 4°C was applied for live-dead discrimination. For intracellular staining, cells were fixed and permeabilized using Cytofix/Cytoperm (BD Biosciences) according to manufacturer’s instructions, washed and resuspended in PBS/1% FCS/0.1% saponin. For intracellular staining and cell surface staining, cells were labeled for 30 min at 4°C with fluorophore-conjugated antibodies (all BD, if not stated otherwise) and washed twice. Flow cytometric analyses were performed on a FACS LSRII (BD Biosciences). Data were analyzed using the FlowJo software (Tree Star Inc., USA). Antibodies: CD11c (HL3)-APC, CD11c (HL3)-PE-Cy7, CD4 (RM4-5)-BV605, CD45 (30-F11)-APC-Cy7, CD45R (RA3-6B2)-PE, CD64 (X54-5/7.1)-PE, Foxp3 (MF23)-AF647, Foxp3 (MF23)-BV421, GATA3 (L50-823)-PE-Cy7, IFNγ (XMG1.2)-PE-Cy7, IFNγ (XMG1.2)-APC, IFNγ (XMG1.2)-FITC, IκBζ (LK2NAP)-PerCP-EF710 (Thermo Fisher Scientific), IL-10 (JES5-16E3)-BV510, IL-10 (JES5-16E3)-FITC (BioLegend), IL-17A (TC11-18H10)-PE, IL-17A (TC11-18H10)-APC-Cy7, IL-4 (11B11)-PE, LY6G/C (RB6-8C5)-PE, I-A/I-E (MHC II) (AF6-120.1)-APC, I-A/I-E (MHC II) (AF6-120.1)-BV421, RORγt (Q31-378)-BV421, and T-bet (4B10)-BV421 (BioLegend).

### Statistics

Statistical analysis of the data was performed with the GraphPad Prism 8 Software. Data were tested for normality using the Shapiro-Wilk normality test. Statistical analyses were then performed *via* unpaired student’s t test or ANOVA for normally distributed data and Mann-Whitney or Kruskal-Wallis multiple comparison test for nonparametric statistics. Statistical significance: *p < 0.05, **p < 0.01, ***p < 0.001, ****p < 0.0001. Error bars represent + standard deviation (SD).

## Results

### Expression of IκBζ Promotes Intestinal Homeostasis in a Mouse Model of Acute Colitis

**Figure 1:**
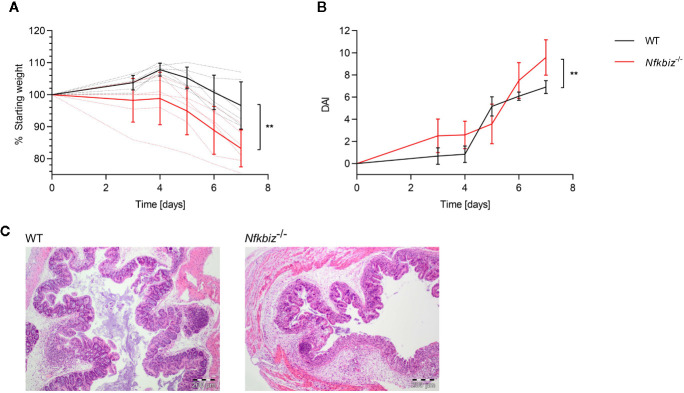
IκBζ expression promotes intestinal homeostasis in the mouse model of DSS colitis. Wild type (WT) (n = 6) and *Nfkbiz* ^-/-^ (n = 6) specific-pathogen free (SPF) mice were administered 2.5% DSS (w/v) in drinking water for 7 days to induce colitis. **(A)** Changes in body weight were monitored throughout the experiment: dotted lines indicate each individual, and continuous lines indicate group means ± SD. **(B)** Disease activity index (DAI) was determined according to the criteria mentioned in the material and methods part, with indicated group means ± SD. **p < 0.005 **(C)** Representative H&E stained colon sections.

To confirm the role of IκBζ in the modulation of mucosal immune responses, we analyzed the impact of IκBζ-expression on the course of intestinal inflammation in a mouse model of dextran sodium sulfate (DSS) –induced acute colitis. Wild type (WT) and *Nfkbiz* ^-/-^ specific-pathogen-free mice were administered 2.5% DSS for seven days in order to induce acute colitis. The severity of disease was estimated by monitoring the weight of the mice and determining the disease activity index (DAI). *Nfkbiz* ^-/-^ mice were found to be significantly more susceptible to DSS colitis, as shown by a significantly increased weight loss and DAI, as well as clear signs of severe colitis as shown by histopathological examination of colon sections (**Figure 1**). Based on these results, we conclude that IκBζ plays an important role in maintaining intestinal homeostasis.

### IκBζ Expression in BMDCs and Intestinal Epithelial Cells Is Differentially Modulated by Distinct Commensals

**Figure 2:**
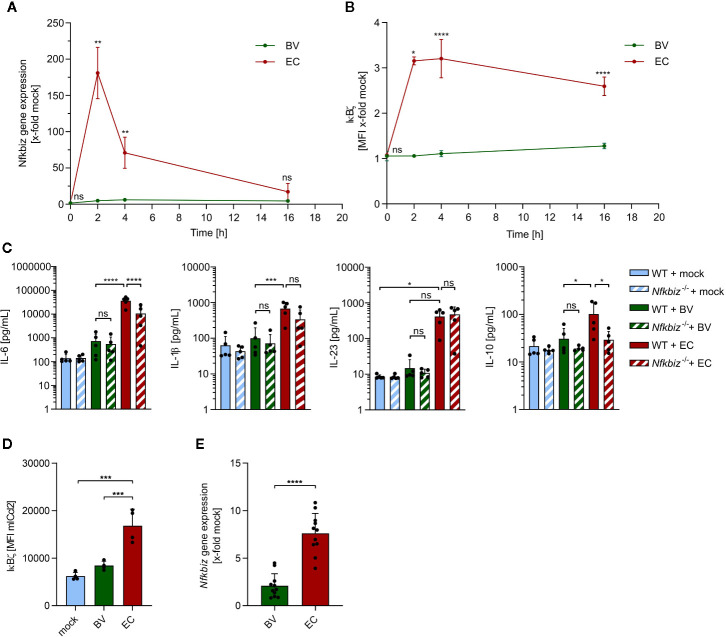
The influence of *B*. *vulgatus* and *E. coli* stimulation on IκBζ expression in bone marrow-derived dendritic cells (BMDCs) and mICcl2 cells. **(A)** *Nfkbiz* gene expression after stimulation of wild type (WT) BMDCs with *B*. *vulgatus* (BV) or *E. coli* (EC) at the indicated time points for a total of 16h as determined by RT-PCR (n = 4). **(B)** The respective IκBζ protein levels were determined by flow cytometry analysis. **(C)** Cytokine secretion into cell culture supernatants of WT BMDCs and *Nfkbiz* ^-/-^ BMDCs after 24 h stimulation with mock, BV and EC was determined by ELISA (n = 5). **(D)** IκBζ protein levels after 2 h stimulation of mICcl2 cells with mock, BV or EC was determined by flow cytometry and **(E)** *Nfkbiz* gene expression after 4 h stimulation of mICcl2 cells with mock, BV or EC was determined by RT-PCR. Data are represented as geometric mean + SD, ns, not significant, *p < 0.05, **p < 0.005, ***p < 0.0005, ****p < 0.00005.

Next, we assessed the contribution of two model gut commensals to IκBζ-dependent activation and maturation of DCs. The mouse gut commensal *B. vulgatus* exhibits low immunogenicity and induces smDCs in the colonic lamina propria (cLP), thus contributing to the promotion of homeostasis and prevention of intestinal inflammation in mouse models for colitis (8, 23, 33). *E. coli*, however, is strongly immunogenic and provokes a pro-inflammatory immune response by inducing mDCs, resulting in intestinal inflammation in *Il-2* deficient mice (8, 23, 34). Since bone marrow-derived dendritic cells (BMDCs) are phenotypically similar tointestinal lamina propria DCs (26) and can be generated in high numbers and comparable maturation status, we used BMDCs to evaluate IκBζ induction by *B. vulgatus* or *E. coli*. Wild type (WT) BMDCs were stimulated with either of the two commensals at a MOI of 1 for 16 h, and *Nfkbiz* gene expression as well as IκBζ protein levels were determined at different time points (**Figures 2A, B**, and **Supplementary Figure 1**). *E. coli* stimulation strongly induced *Nfkbiz* gene expression with a maximal expression at 2 h post stimulation, followed by a decrease over time to levels close to the starting ones (**Figure 2A**). In contrast, stimulation of WT BMDCs with *B. vulgatus* did not significantly alter the basal levels of *Nfkbiz* gene expression. In agreement with the enhanced mRNA levels, 2 h stimulation of WT BMDCs with *E. coli* strongly increased the IκBζ protein levels in comparison to those in *B. vulgatus*-stimulated BMDCs, which did not differ much from the basal protein levels (**Figure 2B**). Yet, the IκBζ protein levels in *E. coli-*stimulated BMDCs did not decrease as strongly and rapidly as the mRNA levels, indicative of a stable protein. These results suggest that IκBζ expression in BMDCs is differently regulated by commensals, with *E. coli* provoking a strong cell response and *B. vulgatus* a weak one. Hence, the question arises whether IκBζ-mediated cytokine secretion required for T cell polarization is also influenced by commensals.

To address this, we measured the secreted levels of Th17-inducing cytokines IL-6, IL1β and IL-23 as well as anti-inflammatory IL-10 in cell culture supernatants of BMDCs derived from WT and IκBζ-deficient (*Nfkbiz^-/-^*) mice after stimulation (**Figure 2C**). IL-6 and IL-1β are crucial for the induction of RORγt, whereas IL-23 is required for Th17 effector functions, since the receptor for IL-23 (IL-23R) is absent on naïve T cells (35). Upon 24 h-stimulation with *E. coli*, but not with *B. vulgatus*, WT BMDCs secreted significantly higher amounts of IL-6, IL-1β and a clearly higher amount of IL-23 than unstimulated WT BMDCs. In agreement with our previous findings (34), *E. coli* stimulation also significantly enhanced IL-10 secretion by WT BMDCs when compared to stimulation with *B. vulgatus*. Cytokine secretion in *B. vulgatus*-stimulated WT BMDCs was generally very low and did not significantly differ from that in unstimulated WT BMDCs. However, IL-6 and IL-10 secretion by *Nfkbiz^-/-^* BMDCs stimulated with *E. coli* was significantly lower than that in WT BMDCs, indicating that IL-6 and IL-10 production by BMDCs is dependent on IκBζ. In contrast, deficiency of IκBζ did not significantly reduce cytokine secretion in *B. vulgatus*-stimulated or unstimulated BMDCs. Hence, IκBζ-mediated cytokine secretion by BMDCs seems to be dependent on a strong stimulus, as provided by *E. coli*.

Since DCs are not the only cell type in the gut expressing IκBζ and in direct contact to the microbiota, we also analyzed commensal-mediated effects on IκBζ-expression in mouse intestinal epithelial cells. We stimulated immortalized mouse small intestinal epithelial cells (mICcl2) cells with PBS (mock), *B. vulgatus* or *E. coli* for 2 and 4 h, and measured IκBζ protein and mRNA levels, respectively. Flow cytometry analysis revealed that, similar to what was observed in BMDCs, IκBζ protein levels were significantly higher in *E. coli*-stimulated cells compared to unstimulated or *B. vulgatus*-stimulated cells after 2 h (**Figure 2D** and **Supplementary Figure 2**). After 4 h, a strong induction of *Nfkbiz* gene expression could still be observed in *E. coli*-stimulated, but not *B. vulgatus*-stimulated cells (**Figure 2E**).

These results indicate that commensals display similar immunogenic effects on different cell types of the gut barrier, facilitating a uniform and coordinated immune response by different cell types.

### Commensals Trigger Secretion of Th17-Inducing Cytokines in BMDCs *via* TLR4 Signaling

**Figure 3:**
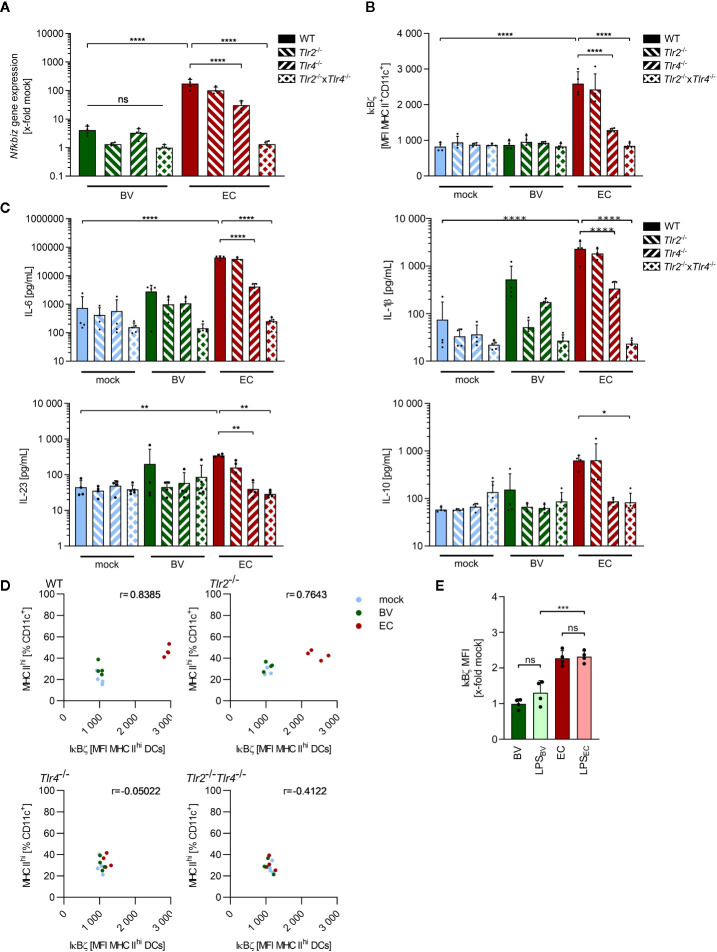
IκBζ induction in bone marrow-derived dendritic cells by commensals is mainly TLR4-dependent. **(A)** *Nfkbiz* gene expression in wild type (WT), *Tlr2* ^-/-^, *Tlr4* ^-/-^ and *Tlr2* ^-/-^x *Tlr4* ^-/-^ BMDCs after stimulation with *B. vulgatus* (BV) or *E*. *coli* (EC) as determined by RT-PCR. **(B)** The respective IκBζ protein levels were determined by flow cytometry after 2 h of stimulation. **(C)** Cytokine secretion into cell culture supernatants after 16 h of stimulation was determined by ELISA. **(D)** Correlation between percentages of MHC II^hi^-expressing BMDCs after 16 h of stimulation and the IκBζ levels after 2 h with indicated Pearson r **(E)** IκBζ levels in WT BMDCs after stimulation with BV, LPS_BV_ (100 ng/ml), EC, LPS_EC_ (100 ng/ml) for 2 h, normalized to IκBζ levels in unstimulated WT BMDCs. Data represent geometric mean + SD, ns, not significant, *p < 0.05, **p < 0.005, ***p < 0.0005, ****p < 0.00005.

As previously described, immunogenicity of the model commensal bacteria *B. vulgatus* and *E. coli* is mainly mediated by their lipopolysaccharide (LPS) and affects both the maturation status and cytokine secretion of BMDCs (26, 34). To identify the bacterial MAMP and the host TLR responsible for the observed IκBζ induction, *Nfkbiz* gene expression, IκBζ protein and secreted cytokine levels were determined in stimulated BMDCs isolated from WT as well as TLR2 (*Tlr2* ^-/-^), TLR4 (*Tlr4* ^-/-^) and TLR2/TLR4 (*Tlr2* ^-/-^ × *Tlr4* ^-/-^) deficient mice. The significant reduction in *Nfkbiz* gene expression and IκBζ protein levels in *Tlr4* ^-/-^ and *Tlr2* ^-/-^ × *Tlr4* ^-/-^ BMDCs, but not *Tlr2* ^-/-^ BMDCs, suggested that the TLR4 ligand LPS was mainly responsible for the high IκBζ induction in *E. coli*-stimulated WT BMDCs (**Figures 3A, B**). In *Tlr2* ^-/-^ × *Tlr4* ^-/-^ BMDCs, the induced levels were even slightly but not significantly lower than those of single knockouts *Tlr2* ^-/-^ and *Tlr4* ^-/-^ BMDCs, suggesting a synergistic effect of TLR2 and TLR4 signaling upon strong immunogenic stimulation. Deficiency of TLR2 and/or TLR4 did not significantly influence IκBζ induction in *B. vulgatus*-stimulated BMDCs, emphasizing the low immunogenicity of this commensal. TLR4 signaling was also responsible for the secretion of Th17-inducing cytokines, since the amount of secreted IL-6, IL-1β, IL-23 and IL-10 was significantly reduced in *Tlr4* ^-/-^ BMDCs despite a strong stimulus, and slightly but not significantly lower in TLR2 and/or TLR4-deficient BMDCs stimulated with weakly immunogenic *B. vulgatus* (**Figure 3C**).

To evaluate the role of IκBζ in TLR-dependent DC maturation, we correlated the percentage of highly mature BMDCs, as indicated by MHC II^hi^ expression, 16 h post stimulation with the IκBζ protein levels measured 2 h after stimulation in these cells (**Figure 3D**). A positive correlation could be observed which decreased upon deficiency for TLR2 and/or TLR4, suggesting a possible role for IκBζ in TLR-ligand induced maturation processes of BMDCs.

To confirm LPS as the main trigger for IκBζ expression, WT BMDCs were stimulated for 2 h with *B. vulgatus*, *E. coli* and the respective LPS (LPS_BV_ and LPS_EC_). As expected, IκBζ protein levels normalized to levels in unstimulated BMDCs did not significantly differ between *B. vulgatus* and LPS_BV_ as well as between *E. coli* and LPS_EC_ (**Figure 3E**). Furthermore, LPS_EC_-induced IκBζ levels were significantly higher than the LPS_BV_-induced protein levels, mirroring the results obtained with *B. vulgatus* and *E. coli* stimulation. This data suggests that the immunogenicity-dependent effects of *B. vulgatus* and *E. coli* on IκBζ expression, cell maturation and cytokine secretion are mediated by their LPS.

### The Unique Composition of the Cytokine Milieu in Response to Various Commensals Differentially Polarizes T Cells

**Figure 4:**
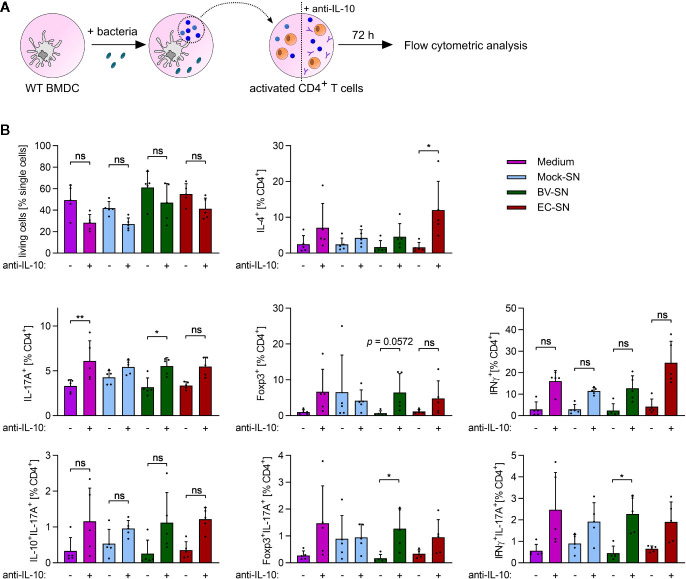
*In vitro* CD4^+^ T cell polarization in response to the supernatant of BMDCs. **(A)** Overview of the experimental setup: WT BMDCs were stimulated with PBS (mock), *B*. *vulgatus* (BV) or *E. coli* (EC) for 16 h. The resulting supernatants were sterile-filtrated and used for differentiation of anti-CD3/anti-CD28-activated WT CD4^+^ T cells with or without addition of neutralizing anti-IL-10 antibodies (10 µg/ml). After 72 h, T cells were analyzed by flow cytometry. **(B)** Flow cytometry analysis of differentiated CD4^+^ T cell subsets after incubation with T cell medium or supernatants of mock, BV or EC-stimulated WT BMDCs with or without addition of neutralizing anti-IL antibodies (10µg/ml) (n = 5). Data represent geometric mean + SD, ns, not significant, *p < 0.05, **p < 0.005.

Antigen-inexperienced, i.e., naïve, CD4^+^ T cells can differentiate into multiple lineages upon activation, depending on the local environment mainly defined by the composition and concentration of the available cytokines (36, 37). As we observed a distinct cytokine secretion pattern in response to *B. vulgatus* and *E. coli*, we analyzed the influence of the different cytokine milieu on CD4^+^ T cell differentiation. To this aim, we antigen-independently activated naïve CD4^+^ T cell with plate-bound anti-CD3 and soluble anti-CD28, and defined their polarization fate in response to sterile-filtrated cell culture supernatant (SN) of BMDCs previously stimulated for 16 h with PBS (mock SN), *B. vulgatus* (BV SN), or *E. coli* (EC SN) (**Figure 4A** and **Supplementary Figure 3**). As control, differentiation in presence of the sole BMDC medium was performed.

To mimic an imbalance between pro- and anti-inflammatory cytokines as reported for the pathogenesis of autoimmune diseases (38), we added neutralizing anti-IL-10 antibody to the cell culture supernatants (medium + anti-IL-10, mock SN+ anti-IL-10, BV SN + anti-IL-10, EC SN + anti-IL-10). Neutralization of extracellular IL-10 appeared to slightly reduce T cell survival after 72 h of incubation (**Figure 4B**). Yet, it also induced a more pronounced differentiation of naïve T cells into Th1 (IFNγ+CD4^+^) and Th2 (IL-4^+^CD4^+^) effector helper subsets. Furthermore, it significantly increased differentiation into Th17 cells (IL-17^+^ CD4^+^) when present alone (BMDC medium only) and in combination with BV SN or EC SN. Yet, BV SN and EC SN induced similar levels of Th17 cells, under both balanced and imbalanced cytokine conditions.

Th17 cells are known to have certain plasticity. On the one hand, they are able to convert to Th1-like Th17 cells, co-expressing IL-17 and IFNγ, and contributing to increased inflammatory activity (39). On the other hand, anti-Th17 Treg cells co-expressing IL-17 and Foxp3 were shown to suppress CD4^+^ T cell proliferation, and found in the inflamed intestinal mucosa of patients with Crohn´s Disease (40). Th17 cells co-expressing IL-17 and IL-10 are instead protective and prevent the accumulation and activity of inflammatory Th17 at sites of inflammation (41). Therefore, we further characterized differentiated Th17 cells with respect to the co-expression of IL-17 with IFNγ, Foxp3 or IL-10 to define their inflammatory or non-inflammatory potential. No significant influence of the differentiation environments on the subsets of Th17 cells was observed (**Figure 4B**, bottom panels). However, the percentage of Foxp3^+^ IL-17^+^ T cells and IFNγ^+^ IL17^+^ T cells significantly increased upon neutralization of IL-10 in BV SN, suggesting that, even in absence of anti-inflammatory IL-10, a balanced Th17 immune response is guaranteed by an increased number of anti-Th17 Tregs.

### *E. coli* Promotes a Pro-Inflammatory CD4^+^ T Cell Response in the Mouse Model of T Cell Transfer Colitis

**Figure 5:**
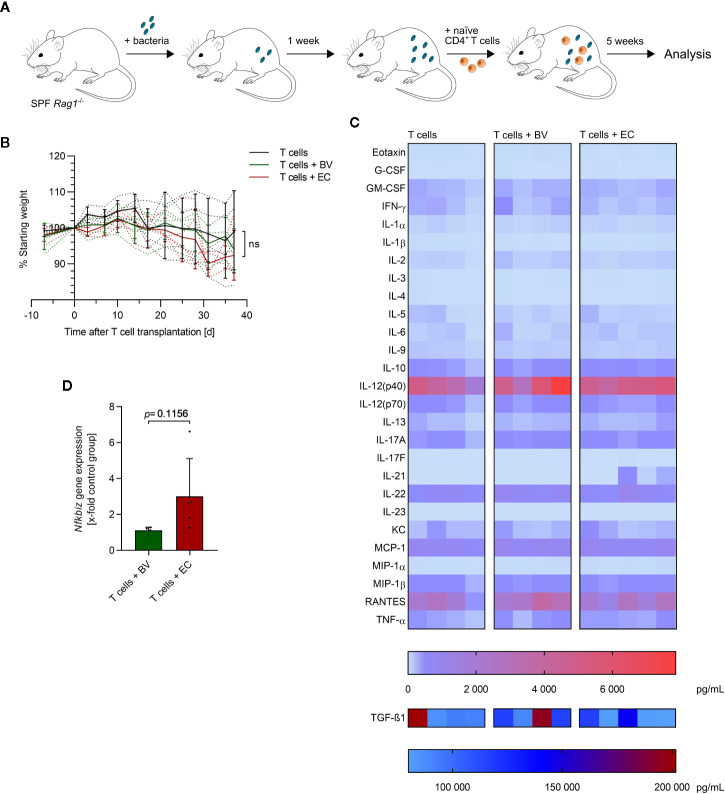
*E. coli*-administration accelerates colitis induction in a T cell transfer model of colitis. **(A)** Overview of the experimental setup: specific-pathogen free (SPF) *Rag1^-/-^* mice were continuously administered with *B*. *vulgatus* (n = 4) or *E*. *coli* (n = 5) *via* drinking water. A control group was left untreated (n = 4). After one week of bacterial association, naïve CD4^+^ T cells were transplanted. Mice were sacrificed 5 weeks after T cell transplantation. **(B)** The change in bodyweight was monitored throughout the experiment: dotted lines indicate each individual and continuous lines indicate group means ± SD, ns, not significant. **(C)** The concentration of the indicated cytokines was determined in mouse serum, using Bio-Plex assays. Each column represents one individual. **(D)** RNA was isolated from colonic tissue and *Nfkbiz* gene expression was determined by RT-PCR. Data represent geometric mean + SD.

The initial lack of Tregs and induction of inflammatory Th1 and Th17 cells are known to play a role in disease onset in the T cell transfer model of colitis in *Rag1^-/-^* mice (42, 43). Transfer of naïve T cells into these immune-deficient mice lacking functional T cells and B cells induces a chronic colonic inflammation that is largely dependent on the microbiota composition (44). We therefore analyzed the impact of administration of a symbiont or a pathobiont, respectively, on DC responses and T helper cell polarization in the colonic lamina propria (cLP) and mesenteric lymph nodes (mLN). SPF *Rag1^-/-^* mice were administered either *B. vulgatus* or *E. coli* by continuous administration of 2 × 10^8^ bacteria per mL drinking water, starting one week prior to transplantation of 5 × 10^5^ naïve T cells (**Figure 5A**). Mice were weighed and drinking water renewed twice a week. Mice were sacrificed five weeks after T cell transplantation.

Administration of *B. vulgatus* or *E. coli* did not lead to significant differences in weight loss over time (**Figure 5B** and **Supplementary Figure 4**). However, a slightly accelerated weight loss was observed in *E. coli*-administered mice starting three weeks after T cell transplantation compared to mice administered with either *B. vulgatus* or no bacteria. Furthermore, a high variation within experimental groups was observed, as indicated by large standard deviations in **Figure 5B**. To evaluate the influence of *B. vulgatus-* and *E. coli-*administration on systemic inflammation, the concentration of serum cytokines was determined (**Figure 5C**). No significant differences were observed between the different experimental groups with the exception of increased IL-21 levels in some *E. coli*-administered mice. Serum concentrations of Th17-inducing IL-6 and IL-1β were very low with IL-23 concentrations even under the detection limit in all mice. Concentrations of the anti-inflammatory IL-10 did positively correlate with concentrations of many pro-inflammatory cytokines (**Supplementary Figure 5**), indicating a systemic repressive function. Yet, *Nfkbiz* gene expression in colonic tissue was found to be higher upon *E. coli*-administration, compared to *B. vulgatus*-administration, giving first hints of a more pronounced Th17 response to a microbiota rich in *E. coli* (**Figure 5D**).

**Figure 6:**
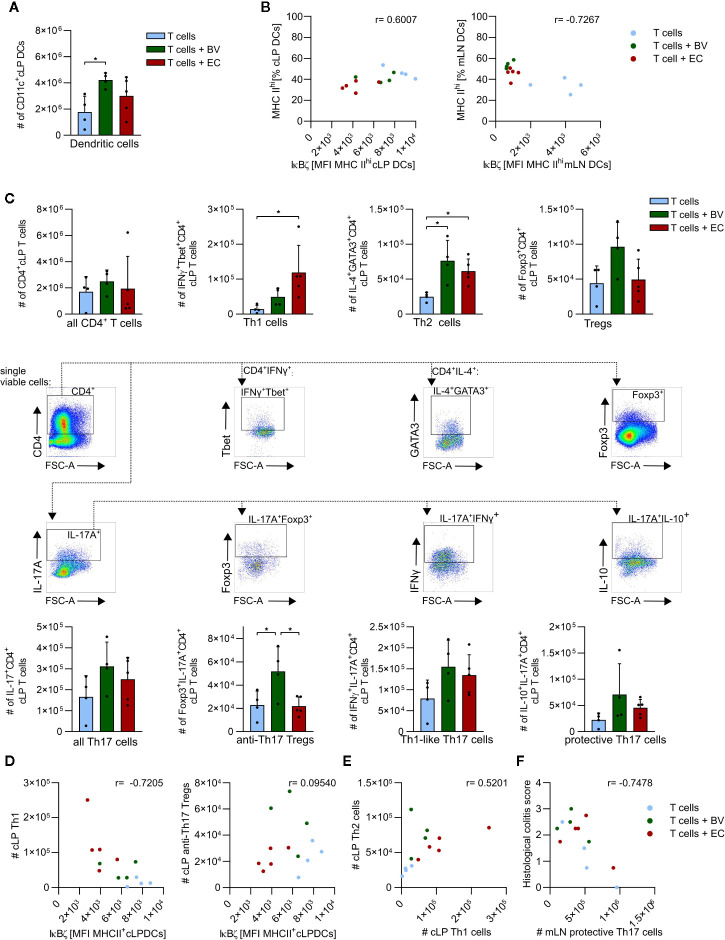
*B. vulgatus* promotes induction of immune regulative mechanisms in a T cell transfer model of colitis. Dendritic cells (DCs) and CD4^+^ T cells were isolated from the colonic lamina propria (cLP) and mesenteric lymph nodes of T cell transplanted *Rag1* ^-/-^ mice and analyzed by flow cytometry (see Fig.5) **(A)** Total number of cLP CD11c^+^ DCs as determined by flow cytometry. **(B)** Correlation between percentages of MHC II^hi^ expressing cLP (left panel) and mLN DCs (right panel) and their IκBζ levels, indicated Pearson r. **(C)** CD4^+^ T cell phenotypes in cLP with corresponding gating. **(D)** Correlations between IκBζ expression in cLP DCs and cLP Th1 cells or CLP anti-Th17 Tregs are indicated Pearson r. **(E)** Th1/Th2 balance in cLP. **(F)** Correlation between histological colitis score and number of cLP protective Th17 cells, as indicated Pearson r. Data represent geometric mean + SD, *p < 0.05.

Flow cytometry analyses revealed that total numbers of cLP DCs were significantly higher in the *B. vulgatus*-administered group compared to the control group, whereas *E. coli*-administration resulted in only slightly increased numbers (**Figure 6A** and **Supplementary Figure 6**). A positive correlation between the maturation status of cLP DCs, as indicated by MHC II^hi^ expression, and IκBζ protein levels in these DCs was observed, with only low percentages of highly mature DCs in *B. vulgatus* or *E. coli*-administered mice (**Figure 6B**, left panel). However, highly mature DCs with low IκBζ levels were observed in mLN of *B. vulgatus* or *E. coli*-administered mice (**Figure 6B**, right panel). In the control group, the percentage of IκBζ^hi^ MHC II^hi^ mLN DCs remained low.

Total numbers of recruited cLP CD4^+^ T cells were not found to be dependent on microbiota composition (**Figure 6C**). Yet, the polarization of these CD4^+^ T cells seemed to be conditioned by microbiota composition: enhanced abundance of *B. vulgatus* clearly induced more Tregs than *E. coli*-administration or no microbiota-manipulation. Also the total numbers of Foxp3^+^ IL-17^+^ anti-Th17 Tregs were significantly higher, and total numbers of IL10^+^ IL-17^+^ protective Th17 cells were slightly higher in *B. vulgatus*-administered mice compared to the other groups. In contrast, *E. coli*-administration resulted in significantly higher total numbers of Th1 cells and slightly higher total numbers of Th1-like Th17 cells than the control group, but at levels similar to those detected in the *B. vulgatus*-administered group. Total numbers of Th2 cells were significantly higher in *B. vulgatus*- or *E. coli*-administered groups compared to the control group. Taken together, polarization of CD4^+^ T cells in the cLP is tilted to rather anti-inflammatory and regulatory phenotypes in *B. vulgatus*-administered mice whereas in *E. coli*-administered mice, cLP CD4^+^ T cells express rather pro- inflammatory markers, promoting colonic inflammation.

In order to evaluate the role of DCs in the induction of the observed phenotypes and overall disease progression, we correlated IκBζ-expression in cLP DCs with CD4^+^ T cell phenotypes in cLP and mLN (**Figure 6D**). IκBζ-expression in cLP DCs was negatively correlated with total numbers of cLP Th1 cells in all experimental groups (**Figure 6D**, left panel). Furthermore, induction of the anti-Th17 Tregs seemed to be dependent on intermediate IκBζ-levels as observed in the *B. vulgatus*- administered group (**Figure 6D**, right panel). High and low IκBζ-levels in cLP DCs in control or *E. coli*-administered mice did not correlate with high numbers of anti-Th17 Tregs.

With respect to the classical Th1/Th2 balance, a shift towards autoimmune-disease promoting Th1 cells was observed in *E. coli*-administered mice whereas in *B. vulgatus*- administered mice the number of Th2 cells exceeded the number of Th1 cells (**Figures 6C, E**) (45). Consistently, the grade of colonic inflammation negatively correlated with the induction of protective Th17 cells in mLN, emphasizing the anti-inflammatory role of these cells (**Figure 6F**).

### Increased Abundance of *B. vulgatus* in Microbiota Dampens the Secretion of Pro-Inflammatory Cytokines by BMDCs

**Figure 7:**
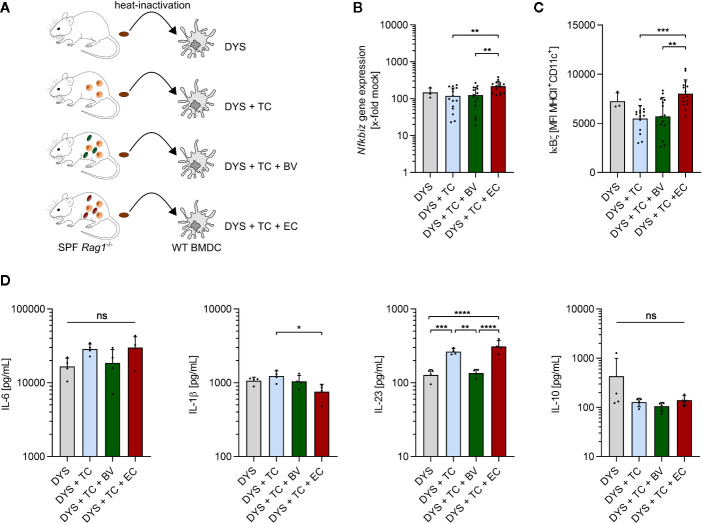
Enhanced abundance of *E. coli* increases IκBζ levels in dendritic cells. **(A)** Overview of the experimental setup: fecal samples were collected from SPF *Rag1^-/-^* mice prior to bacterial administration and T cell transplantation (DYS) as well as from T cell transplanted mice with established colitis, and left untreated (DYS + TC) or administered with *B*. *vulgatus* (DYS + TC + BV) or *E*. *coli* (DYS + TC + EC) *via* drinking water. Samples were dissolved in PBS, heated for 15 min at 80°C, filtered and used in a concentration of 100 µg/ml for a 2 h and 4 h stimulation of wild type (WT) bone marrow-derived dendritic cells (BMDCs). **(B)** *Nfkbiz* gene expression, as determined by RT-PCR and **(C)** IκBζ protein levels, as determined by flow cytometry, after 2 h stimulation of WT BMDCs. **(D)** Secreted cytokines after 4 h stimulation of WT BMDCs, as determined by ELISA. Data represent geometric mean + SD, ns, not significant, *p < 0.05, **p < 0.005, ***p < 0.0005, ****p < 0.00005.

The above presented results indicate that the enhanced intestinal abundance of *B. vulgatus* leads to an increase in regulatory/anti-inflammatory CD4^+^ T cell subsets whereas higher numbers of *E. coli* promote differentiation of pro-inflammatory CD4^+^ T cells in an immune-compromised host with a presumably dysbiotic microbiota. However, a differential activation of DCs by the two commensals could not be clearly observed in these mice. To directly link our *in vitro* results with those obtained in the model of T cell transfer colitis, we collected fecal samples from representative *Rag1^-/-^* mice with a presumably dysbiotic microbiota (DYS) prior to bacterial administration and from T cell transplanted mice with or without commensal enrichment after development of colitis (DYS + TC, DYS + TC + BV, DYS + TC + EC). Heat-inactivated fecal samples were then used to stimulate WT BMDCs (**Figure 7A**). Increased abundance of *E. coli* induced significantly higher *Nfkbiz* gene expression (**Figure 7B**) and IκBζ–protein levels (**Figure 7C**) than all the other microbiota. However, we did not observe a significant increase in the secretion of pro-inflammatory cytokines in response to DYS + TC + EC- stimulation as compared to DYS+TC (**Figure 7D**). Rather, 4 h stimulation of BMDC with DYS + TC + BV decreased the secretion of pro-inflammatory cytokines, with a slight but not significant decrease of secreted IL-6, and a significant lower secretion of IL-23 compared to DYS + TC or DYS + TC + EC. The levels of IL-10 decreased as a result of T cell transfer.

## Discussion

The impact of the intestinal microbiota on health and disease is indisputably large. Due to the close link between microbiota and host immunity, it is not surprising that dysbiosis is associated with many diseases linked to a malfunctioning immune system, e.g., autoimmune diseases. A local impact of a disturbed microbiota is well described for inflammatory bowel diseases (IBD) such as Crohn’s disease or ulcerative colitis). Moreover, many extra-intestinal diseases such as type 1 diabetes, rheumatoid arthritis, asthma or multiple sclerosis have been reported to be influenced by the microbiota (3, 46).

The first model commensal used in this study, *B. vulgatus*, belongs to the phylum of Bacteroidetes, one of the most abundant phyla in the mammalian gut, and was found to reduce inflammation in mouse models of colitis (23, 47). A decrease in *Bacteroides* species was reported in patients with IBD, together with the simultaneous increase of facultative anaerobes such as *E. coli*, the second model commensal used in this study (48). *E. coli* is a colitogenic pathobiont,that can promote intestinal inflammation in genetically predisposed hosts (23). Differences in bacterial immunogenicity as well as in the interaction with several cell types of the innate and adaptive immune system are accountable for these contrary outcomes. In this study, we focused on the interaction of commensals with DCs and the resulting CD4^+^ T cell response *in vitro* and *in vivo* in an autoimmune-driven mouse model of colitis (49).

The role of IκBζ has already been extensively studied in various autoimmune diseases and cell types. For instance, keratinocyte-derived IκBζ was found to drive psoriasis (50, 51); and mice deficient in IκBζ are resistant to EAE due to a defect in Th17 development, explainable by the fact that IκBζ enhances IL-17 expression by directly binding to the regulatory region of the *Il-17* gene (16). However, IκBζ-deficient epithelial cells provoke a Sjögren’s syndrome-like inflammation in mice, and IκBζ-deficient hepatocytes showed defective proliferation due to impaired TLR4-signaling (21, 52). Furthermore, IκBζ exerts both inhibitory and transcription-promoting effects on NFκB activity. The transcription factor NFκB plays an importantrole in cellular responses to stress, injury and inflammation (53). Its subunits p50, p52, p65 (RelA), RelB and c-Rel can form various homo- and heterodimers which bind to specific DNA elements to induce target gene expression of e.g., IL-6, IL-1β, IL-23, or IL-10 (54–56). More recently, upregulation of *Nfkbiz* has been detected in inflamed intestinal tissue of UC patients, suggesting that an altered function of IκBζ may contribute to the development of the disease (57). We could demonstrate a deleterious effect of IκBζ deficiency in the mouse model of acute DSS-induced colitis (**Figure 1**). *Nfkbiz* ^-/-^ mice progressed towards a significantly more severe disease than WT mice, indicating an important role of IκBζ for intestinal inflammatory responses to DSS administration. Taken together, these data suggest that the function of IκBζ needs to be tightly regulated in many cell types: too much or too little of its activity can lead to disease.

We could also show that bacterial immunogenicity regulates IκBζ expression in DCs thus driving either an inflammation-promoting or tolerogenic DC phenotype. In our previous studies, we demonstrated that *B. vulgatus* induces smDCs, characterized by a lower expression of maturation markers, such as MHC II, CD40, CD80 and CD86, as well as lower secretion of pro-inflammatory cytokines compared to mDCs induced by *E. coli* (8, 58). These smDCs are tolerant towards maturation-inducing stimuli and are unable to induce pro-inflammatory Th1 and Th17 responses (8). Here, we could relate the expression levels of IκBζ to the degree of DC maturation and induction of T cell differentiation*: E. coli*, but not *B. vulgatus* increased the mRNA and protein levels of IκBζ (**Figures 2A, B**). Based on this, we propose that the transition from smDCs to mDCs requires a relief of the tight regulation on IκBζ expression and activity.

IκBζ is generally induced by stimulation with MAMPs or the cytokines IL-1, IL-17 and IL-18 (16, 20, 21, 57). Here, we could demonstrate that *E. coli*-induced IκBζ expression as well as BMDC maturation is mainly mediated by LPS *via* TLR4 signaling (**Figure 3**). Despite being one of the most conserved structures in Gram-negative bacteria, differences in immune-activating activities of LPS have been observed before: isolated LPS_BV_ displayed only weak agonistic interactions with the host MD2/TLR4 receptor complex, thus inducing smBMDCs, whereas isolated LPS_EC_ potently activated the MD2/TLR4 receptor complex, causing rather pro-inflammatory signaling by mBMDCs (26). We additionally demonstrate that the extent of LPS-induced TLR4 signaling impacts the ability of BMDCs to induce a Th17 response: a stronger activation significantly enhances secretion of Th17-promoting cytokines by BMDCs (**Figures 2C** and **3C**). In addition, we observed a synergistic effect of TLR2 and TLR4 signaling upon a strong stimulus, indicated by a decreased response in BMDCs deficient for both receptors compared to BMDCs deficient for only one of these TLRs. This observation supports earlier findings, describing a marked increase in pro-inflammatory cytokine secretion by mouse peritoneal macrophages upon co-stimulation with TLR2 and TLR4 ligands compared to the stimulation of either receptor alone (59). As previously reported, only secretion of IL-6 and IL-10 was found to be IκBζ-dependent (21, 60). IL-6 is mainly induced by p65/p50 NFκB heterodimers and IL-10 by p50/p50 NFκB homodimers (56, 61). IκBζ preferentially associates with p50 present in p65/p50 heterodimers or p50/p50 homodimers, stabilizes promoter binding and thus assists expression of IL-10 and IL-6 (17, 60, 61). IL-1β is mainly induced by subunits p65 and cRel and, thus, presumably not preferentially bound by IκBζ (62). Nevertheless, an indirect influence of IκBζ activity on IL-1β secretion has been elucidated recently: IκBζ upregulates the transcription of the *Nlrp3* gene, which encodes the inflammasome component NLRP3 (63). Activation of the NLRP3 inflammasome leads to the cleavage of inactive pro-IL-1β into active IL-1β, which can then be secreted by the cell. Kim et al. reported that *Nfkbiz* deficiency results in impaired IL-1β secretion, which we could confirm (**Figure 2C**). An IκBζ-dependent regulation of IL-23 secretion by BMDCs was however not observed.

We tested whether the cytokine milieu of stimulated BMDCs is sufficient for determining the differentiation fate of already activated T cells. We could not observe significant induction of effector T cells (Th1, Th2, Tregs, Th17 subsets) (**Figure 4**). A slightly but insignificantly increased survival of T cells could be observed upon differentiation with the cytokine mix originating from *B. vulgatus*- and *E. coli*- stimulated BMDCs. Cytokines such as IL-6 serve as T cell survival factors and are secreted in higher amounts by BMDCs upon contact with bacterial antigens (**Figures 2C** and **3C**) (64). IL-10 is known to exert a critical role in limiting immune-mediated inflammation and to prevent autoimmune pathologies. IL-10 is broadly expressed by many cell types of the innate and adaptive immune system, serving as feedback negative regulator of the innate effector functions of macrophages, DCs and, indirectly, T cells. Furthermore, IL-10 stimulates its own production by enhancing differentiation of IL-10-secreting Tregs (65). Interestingly, creating an “imbalanced” pro-inflammatory cytokine environment by neutralizing IL-10 in the cytokine mixes led to a slightly decreased survival in all conditions tested. Nevertheless, it also increased differentiation of effector T cells. The cytokine mix secreted by *E. coli*–stimulated BMDCs induced significantly higher amounts of Th2 cells upon neutralization of IL-10, suggesting a Th2-inhibiting action of IL-10 upon exposure to strong stimuli. Since overshooting Th2 responses provoke allergic reactions, a strategy for inducing IL-10-secreting DCs with strong stimuli such as bacteria or bacterial components is of large therapeutic interest (66). Furthermore, DC-secreted IL-10 also appears to inhibit Th1 differentiation upon a strong stimulation, here represented by *E. coli-*stimulation. This effect could be abolished by neutralization of IL-10 and was less evident with the other cytokine mixes used. Neutralization of IL-10 in *B. vulgatus-*induced cytokine mixes resulted in significantly increased differentiation of Th17 cells, especially of those expressing Foxp3 and IFNγ. However, the percentage of induced CD4^+^ T cells was relatively low, questioning the biological relevance of the observed differences. Comparatively high amounts of induced Foxp3^+^ Tregs could be observed by cytokine mixes produced by unstimulated immature BMDCs, which was not significantly influenced by IL-10-neutralization. Immature DCs are known to promote T cell anergy and generate Tregs (5). Here, we suggest a Treg-promoting effect by immature DCs independent of antigen presentation and IL-10, which needs further investigation.

When we evaluated the immuno-modulating effects of *B. vulgatus* and *E. coli* under inflammatory conditions in a genetically predisposed host with a presumably dysbiotic microbiota, *E. coli* administration induced colitis slightly but not significantly quicker than an unchanged microbiota or *B. vulgatus*administration as indicated by accelerated weight loss beginning 3 weeks after T cell transfer (**Figure 5**). Nonetheless, flow cytometry analysis of the cLP immune cells revealed significant differences in CD4^+^ T cell subsets even though the absolute numbers of CD4^+^ T cells remained equal: In *B. vulgatus*-administered T cell-transplanted *Rag1* ^-/-^ mice, numbers of regulatory and anti-inflammatory T helper subsets were higher than upon *E. coli* administration or in T cell-transplanted control mice, indicating a potent regulation of inflammation (**Figure 6**). In contrast, *E. coli* administration resulted in high numbers of pro-inflammatory Th1 and Th1-like Th17 cells in the cLP, indicating an uncontrolled inflammation, which was not dampened by low numbers of regulatory CD4^+^ T cell phenotypes. Administration of commensals thus seems to manipulate the Th1/Th2/Th17/Treg balance as well as the pathogenicity of induced Th17 cells. In previous studies, we had already observed that *B. vulgatus* impaired inflammation in T cell-transplanted *Rag1* ^-/-^ mice, whereas transplantation of *Enterobacteriaceae*-rich microbiota strongly exacerbated the course of colitis (27, 33). So far, the cellular mechanisms underlying the protective effect of *B. vulgatus* remained unknown. Here, we could shed light on the influence of the two commensal bacteria on T cell polarization and disease progression.

In addition to the *in vitro* experiments, we observed increased *Nfkbiz* gene expression in inflamed colonic tissue isolated from T cell-transplanted *Rag1* ^-/-^ mice administered with *E. coli*- compared to control or *B. vulgatus*-administered mice. This finding could not be completely traced back to intestinal DCs as sole source of IκBζ-expressing cells. Since mouse small intestinal epithelial cells also increased IκBζ expression upon stimulation with *E. coli in vitro* (**Figures 2D, E**), we assume that the measured *Nfkbiz* expression originated from intestinal epithelial cells.We are aware of the limitation that small intestinal cells do not fully recapitulate the response of colonic tissue and might not be a colonization site for these commensals.

In IBD, the colonic barrier is weakened, resulting in a close contactof the commensals with the epithelial layer (67). This increased contact to epithelial cells represents an antigen-overload, and can lead to an inappropriate and dysregulated response of CD4^+^ T cells, resulting in pro-inflammatory phenotypes and, eventually, in chronic inflammation.

We cannot rule out a contribution of DC-derived IκBζ to the consolidation of inflammation. Intestinal DCs migrate to the mLN upon antigenic challengewhere they present microbiota-derived antigens to naïve T cells thus initiating an adaptive immune response. Recognition of the cognate antigen along with DC-secreted lineage specifying cytokines leads to the differentiation and proliferation of effector T cells, which migrate to the effector site, e.g., the cLP (68, 69). On a first sight, low IκBζ levels observed *in vivo* in cLP and mLN DCs in bacteria-administered T cell-transplanted *Rag1^-/-^* mice would contradict the *in vitro* findings. Ir is however conceivable that cLP and mLN DCs represent DCs in different stages of maturation and differentiation. An earlier accumulation of IκBζ results in suppression of NFκB-induced gene transcription due to the inhibitory activity of IκBζ, creating a self-limiting negative feedback loop. We speculate that the *in vivo*-induced IκBζ expression levels are already diminished in the analyzed cLP and mLN DCs as a result of its self-limitation at a later time point of the maturation stage. WT BMDCs stimulated with heat-inactivated microbiota samples of T cell-transplanted *Rag1* ^-/-^ mice from the experiment discussed above confirmed the commensal-dependent IκBζ expression in DCs: Supporting the inflammation-dampening influence of *B. vulgatus* is the significantly decreased secretion of IL-23 by BMDCs stimulated with the microbiota of *B. vulgatus*-administered mice, a cytokine responsible not only for the maintenance of Th17 cells but also for the innate immune-based pathology (70).

In conclusion, our study suggests that modulating the host’s immune response by commensal bacteria can define the outcome of a Th17-mediated disease, at least in part, *via* regulation of IκBζ in DCs. These findings can be applied for the optimization of microbiota-based therapeutic strategies.

## Data Availability Statement

The original contributions presented in the study are included in the article/**Supplementary Material**. Further inquiries can be directed to the corresponding author.

## Ethics Statement

This study was carried out in accordance with the principles of the Basel Declaration. Protocols and experiments involving mice were reviewed and approved by the responsible Institutional Review Committee and the local authorities within H5/10, H1/15, §4 09.01.2015, §4 14.06.2017 and §4 28.09.2017 approval.

## Author Contributions

LM, AlS, KS-O, and J-SF conceived and designed the experiments. LM, MT, H-CL, JK, CK, AL, AG, AnS, and SM performed the experiments. LM, MT, HC-L, and J-SF analyzed the data. LM, AL, and J-SF wrote the manuscript. All authors contributed to the article and approved the submitted version.

## Funding

This work was funded by the Deutsche Forschungsgemeinschaft (DFG, German Research Foundation) under Germany’s Excellence Strategy—EXC-2124 and Collaborative Research Centres 685 (CRC685), the DFG research training group 1708, the Bundesministerium für Bildung und Forschung (BMBF), and the German Center for Infection Research (DZIF).

## Conflict of Interest

The authors declare that the research was conducted in the absence of any commercial or financial relationships that could be construed as a potential conflict of interest.
