# Supplementary material for: Gut Commensal-Induced IκBζ Expression in Dendritic Cells Influences the Th17 Response
Source: Front Immunol. 2021 Jan 19;11:612336. doi: 10.3389/fimmu.2020.612336 (PMC7851057; doi:10.3389/fimmu.2020.612336)
Supplement: Supplementary file 1 [file Image_1.pdf]

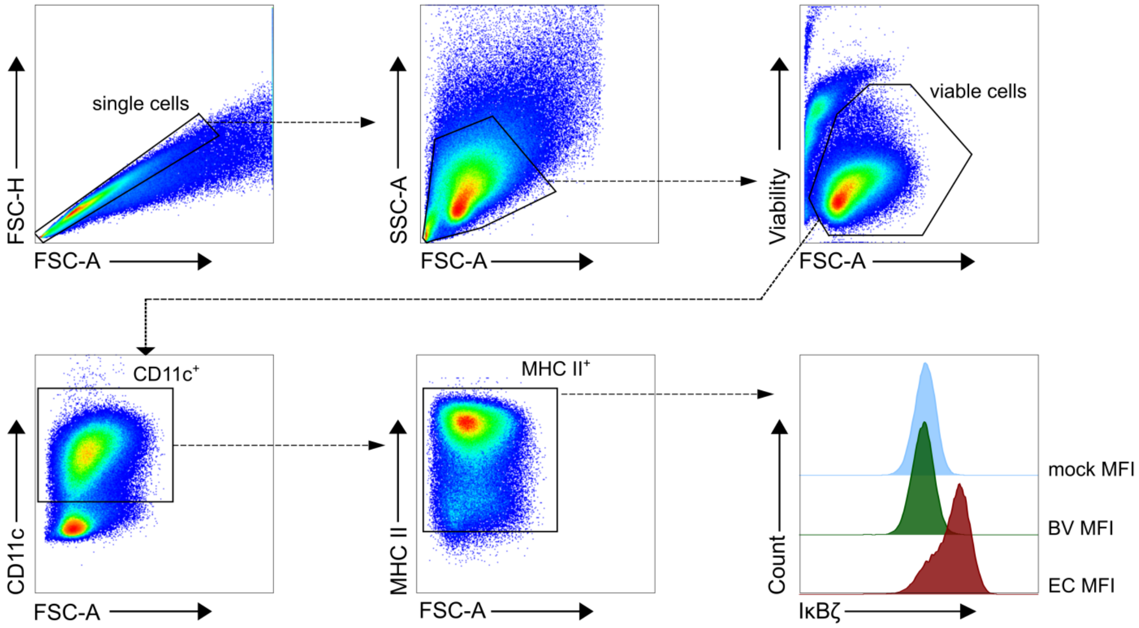

Supplementary Figure 1: Gating strategy applied for the flow cytometry analysis of bone marrow derived dendritic cells (BMDCs). In order to quantify IκBζ protein levels in BMDCs, cell doublets (FSC-A/FSC-H), cell debris (FSC-A/SSC-A) and dead cells (fixable viability dye $^{+}$) were excluded from further analysis. Mean fluorescence of IκBζ was determined in CD11c^{+} MHCII^{+} BMDCs. Here, representative histograms for IκBζ MFI in BMDCs stimulated with PBS (mock), *B. vulgatus* (BV) and *E. coli* (EC) are depicted.
