# Supplementary material for: Gut Commensal-Induced IκBζ Expression in Dendritic Cells Influences the Th17 Response
Source: Front Immunol. 2021 Jan 19;11:612336. doi: 10.3389/fimmu.2020.612336 (PMC7851057; doi:10.3389/fimmu.2020.612336)
Supplement: Supplementary file 2 [file Image_2.pdf]

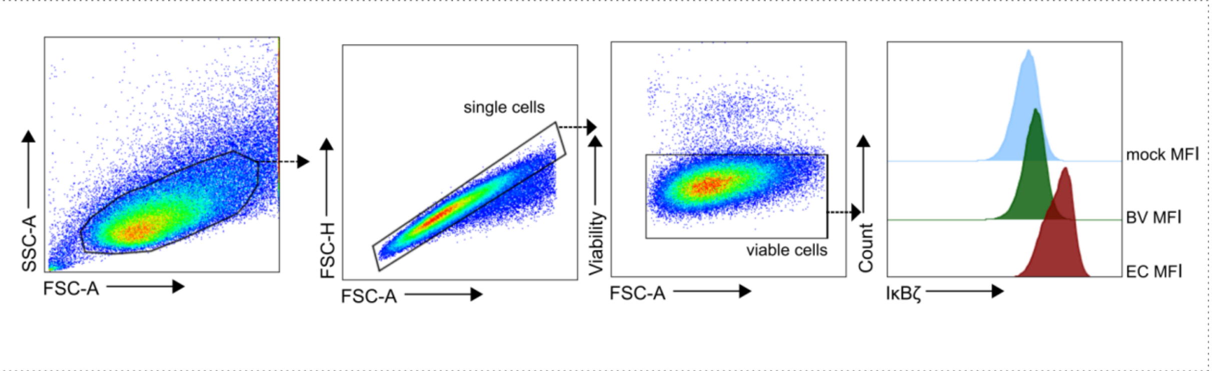

Supplementary Figure 2: Gating strategy applied for the flow cytometry analysis of mICcl2 cells. In order to quantify IκBζ protein levels in mICcl2 cells, cell debris (FSC-A/SSC-A), cell doublets (FSC-A/FSC-H) and dead cells (fixable viability dye $^{+}$) were excluded from further analysis. Mean fluorescence of IκBζ was determined in single, viable cells. Depicted here are representative histograms for IκBζ MFI in mICcl2 cells stimulated with PBS (mock), *B. vulgatus* (BV) and *E. coli* (EC).
