# Supplementary material for: Gut Commensal-Induced IκBζ Expression in Dendritic Cells Influences the Th17 Response
Source: Front Immunol. 2021 Jan 19;11:612336. doi: 10.3389/fimmu.2020.612336 (PMC7851057; doi:10.3389/fimmu.2020.612336)
Supplement: Supplementary file 3 [file Image_3.pdf]

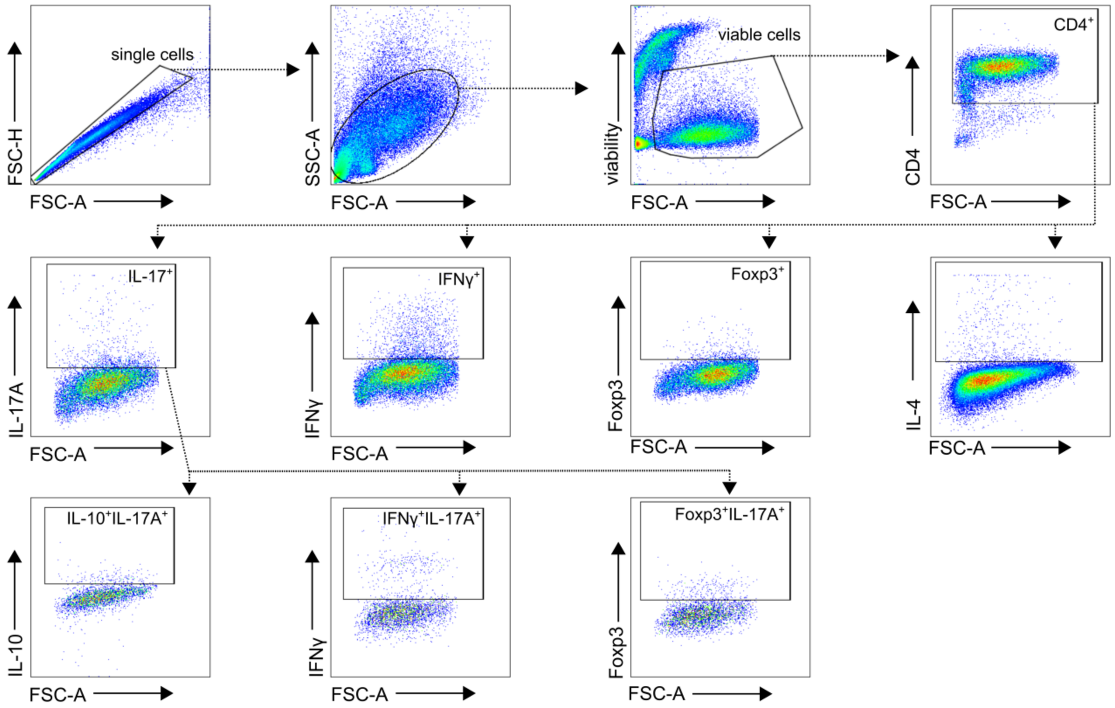

Supplementary Figure 3: Gating strategy applied for the flow cytometry analysis of CD4^{+} T cell subsets. In order to analyze different CD4^{+} T cell subsets, cell doublets (FSC-A/FSC-H), cell debris (FSC-A/SSC-A), and dead cells (fixable viability dye $^{+}$) and CD4^{-} cells were excluded from further analysis. Subsets were determined with specific gates set according to FMO controls.
