# Supplementary material for: Gut Commensal-Induced IκBζ Expression in Dendritic Cells Influences the Th17 Response
Source: Front Immunol. 2021 Jan 19;11:612336. doi: 10.3389/fimmu.2020.612336 (PMC7851057; doi:10.3389/fimmu.2020.612336)
Supplement: Supplementary file 4 [file Image_4.pdf]

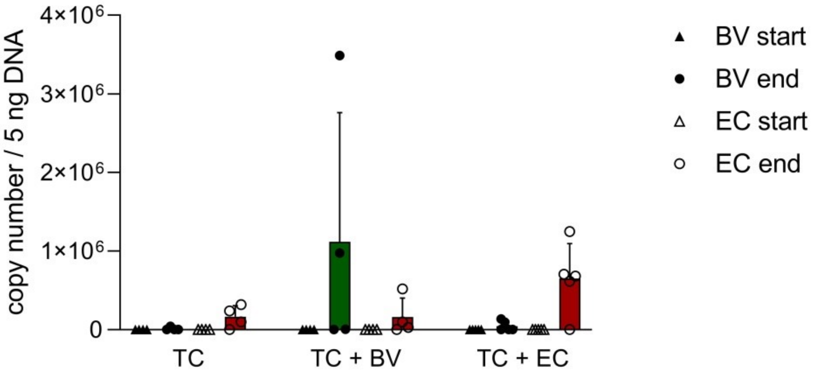

Supplementary Figure 4: Concentration of *B. vulgatus* and *E. coli* in fecal samples of *Rag1*$^{-/-}$ mice. Fecal samples were collected prior bacterial administration (BV start, EC start) and at the end of the experiment (BV end, EC end). Copy numbers of *B. vulgatus*- and *E. coli*-specific 16S rDNA genes were determined from 5 ng of gDNA
