# Supplementary material for: Gut Commensal-Induced IκBζ Expression in Dendritic Cells Influences the Th17 Response
Source: Front Immunol. 2021 Jan 19;11:612336. doi: 10.3389/fimmu.2020.612336 (PMC7851057; doi:10.3389/fimmu.2020.612336)
Supplement: Supplementary file 5 [file Image_5.pdf]

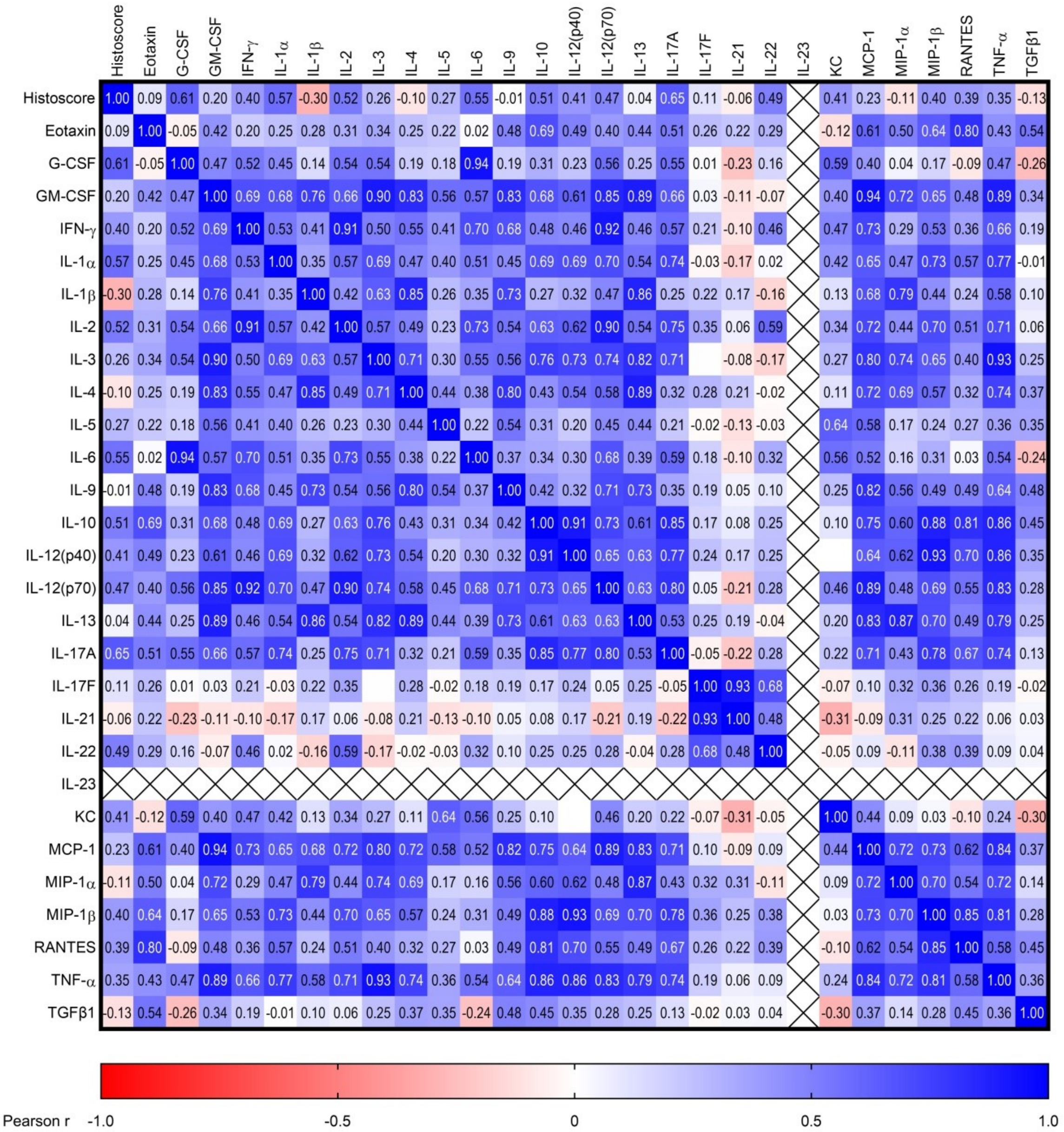

Supplementary Figure 5: Correlation matrix of histological colitis scores and respective serum cytokines of T cell-transplanted *Rag1*$^{-/-}$ mice for the experiment described in Fig. 5. Positive Pearson r value= positive correlation (blue), negative Pearson r value =negative correlation (red). IL-23: no Pearson r calculable since measured concentrations were under the detection limit.
