# Supplementary material for: Gut Commensal-Induced IκBζ Expression in Dendritic Cells Influences the Th17 Response
Source: Front Immunol. 2021 Jan 19;11:612336. doi: 10.3389/fimmu.2020.612336 (PMC7851057; doi:10.3389/fimmu.2020.612336)
Supplement: Supplementary file 6 [file Image_6.pdf]

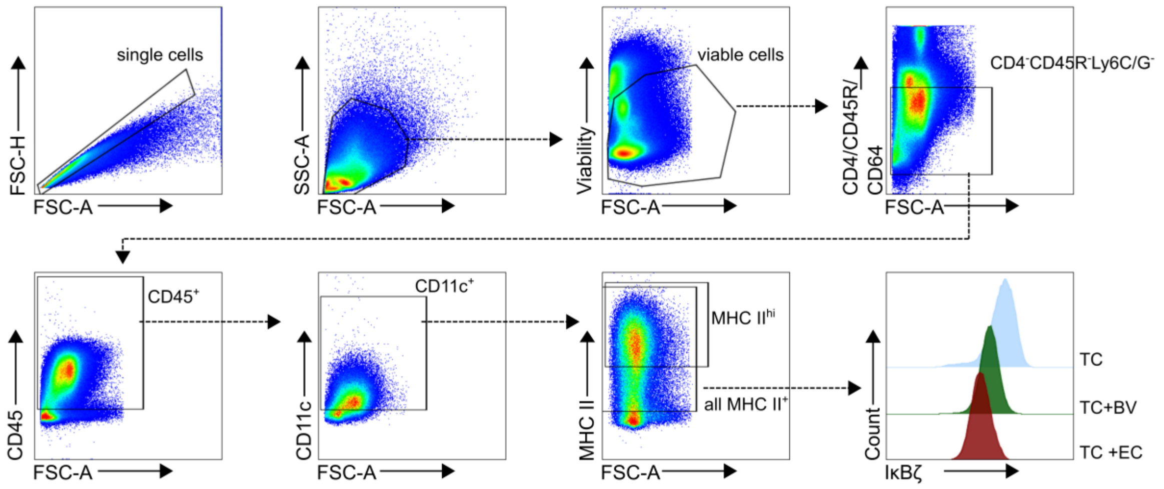


**Supplementary Figure 6: Gating strategy for flow cytometry analysis of cLP and mLN DCs**. Cells were isolated from cLP and mLN of gnotobiotic WT mice or T cell transplanted *Rag1*$^{-/-}$mice from experiments described in Fig. 4 and Fig. 5. In order to quantify IκBζ protein levels in DCs, cell doublettes (FSC-A/FSC-H), cell debris (FSC-A/SSC-A), dead cells (fixable viability dye $^{+}$) and CD4$^{+}$CD45R$^{+}$Ly6C/G$^{+}$ cells were excluded from further analysis. CD45$^{+}$ CD11c$^{+}$ cells were regarded as DCs and mean fluorescence of IκBζ was determined in MHC II$^{+}$ DC and MHC II$^{hi}$ DC populations. Here, representative histograms for IκBζ MFI in MHC II$^{+}$ DCs isolated from T cell transplanted (TC) mice without or with *B. vulgatus* (TC + BV) or *E. coli* (TC + EC) administration are depicted.
